# Syphilis and Venereal Diseases as a Public Health Problem

**Published:** 1919

**Authors:** 


**_Syphilis and Venereal Diseases as a Public Health Problem._** By H. G. Irvine. *Journal of the American Medical Association*, September 28, 1918.

The author emphasizes the danger of making venereal diseases " secret diseases ", in fact as well as in name, instead of handling them along the same lines as other infectious and communicable affections. Since the war, however, the imperative need for early diagnosis, treatment, and control of veneral diseases has been felt, and an adequate program adopted for the army. Moreover, the War Department began to call on the public health officials to undertake definite campaigns against these diseases.

The program for a state divides itself logically into 4 parts, the first 3 being concerned with the control of 3 distinct groups of patients, the 4th with the educational work.

The first group of patients comprises those needing enforced quarantine and treatment : prostitutes, both professional and clandestine, prisoners and inmates of institutions, and incorrigible patients. So far as prostitution is concerned, active co-operation of the police departments is necessary with a view to supressing it or at least strongly reducing it. Arrangements should be made for a proper examination, including Wassermann test of every prisoner, and adequate treatment instituted. If necessary, cases should be quarantined beyond the time of sentence so that no individual in an infectious condition would be allowed his freedom.

The fact that only about 30 to 50 o/o of the patients with syphilis or gonorrhea receive adequate medical service is due to 2 causes : (1) many physicians are not sufficiently trained in the care of these diseases ; (2) many patients cannot afford to pay for sufficient treatment to arrive at a cure. The author insists upon the necessity of opening dispensaries where proper treatment would be given to those who cannot afford any fees. Many such clinics have already been opened in the various States, and Great Britain is now following this program.

Adequate provision must be made for private as well as indigent patients. There should be a system of notification identifying the persons venereally diseased, and providing for a report of the name and address of those who by their conduct are endangering others. The reports would always be strictly confidential. A similar system was adopted with success in California. Rules should be made preventing the sale of any preparation for the treatment of syphilis or gonorrhea, as in a large proportion of cases the patients come to the physician after having tried some treatment recommended by the druggist, which has resulted in loss of time and increased the gravity of the case. The control of patients going to private practitioners depends almost entirely on the co-operation of the medical profession, both individually and collectively.

The systematic education of both laity and physicians, as to the danger of the disease, should be conducted along 2 lines :

(1) A campaign should be undertaken to give information from the disease standpoint. This should comprise lectures, distribution of pamphlets, setting up of placards, etc.

(2) Courses covering biology, psychology of sex, sociology, sex hygiene and venereal diseases should be arranged in the universities and colleges; courses should be given to hospital, public health and school nurses; a course of study on sex education should be planned for parents' and teachers' associations; finally, communities should be stimulated to the further use of present recreational facilities.

Further, people must be made to understand that something can be done if exposure has taken place. Lectures have already been given in the various States in co-operation with the War Department's Commission on Training Camp activities, and pamphlets have been distributed.

A well rounded program for combatting venereal diseases must take cognizance of every factor that enters into their spread; any attempt to limit the work to any one field will fail, as has been proved by previous experience.

